# *Tetrahymena*: An Alternative Model Host for Evaluating Virulence of *Aeromonas* Strains

**DOI:** 10.1371/journal.pone.0048922

**Published:** 2012-11-08

**Authors:** Mao-Da Pang, Xiao-Qin Lin, Meng Hu, Jing Li, Cheng-Ping Lu, Yong-Jie Liu

**Affiliations:** College of Veterinary Medicine, Nanjing Agricultural University, Nanjing, China; University of Malaya, Malaysia

## Abstract

An easier assessment model would be helpful for high-throughput screening of *Aeromonas* virulence. The previous study indicated the potential of *Tetrahymena* as a permissive model to examine virulence of *Aeromonas hydrophila.* Here our aim was to assess virulence of *Aeromonas* spp. using two model hosts, a zebrafish assay and *Tetrahymena*-*Aeromonas* co-culture, and to examine whether data from the *Tetrahymena thermophila* model reflects infections in the well-established animal model. First, virulence of 39 *Aeromonas* strains was assessed by determining the 50% lethal dose (LD_50_) in zebrafish. LD_50_ values ranging from 1.3×10^2^ to 3.0×10^7^ indicated that these strains represent a high to moderate degree of virulence and could be useful to assess virulence in the *Tetrahymena* model. In *Tetrahymena*-*Aeromonas* co-culture, we evaluated the virulence of *Aeromonas* by detecting relative survival of *Aeromonas* and *Tetrahymena*. An *Aeromonas* isolate was considered virulent when its relative survival was greater than 60%, while the *Aeromonas* isolate was considered avirulent if its relative survival was below 40%. When relative survival of *T. thermophila* was lower than 40% after co-culture with an *Aeromonas* isolate, the bacterial strain was regarded as virulent. In contrast, the strain was classified as avirulent if relative survival of *T. thermophila* was greater than 50%. Encouragingly, data from the 39 *Aeromonas* strains showed good correlation in zebrafish and *Tetrahymena*-*Aeromonas* co-culture models. The results provide sufficient data to demonstrate that *Tetrahymena* can be a comparable alternative to zebrafish for determining the virulence of *Aeromonas* isolates.

## Introduction

*Aeromonas* is a Gram-negative bacterium that belongs to the family *Aeromonadaceae* [1]. Recently, the genus *Aeromonas* has received increased attention because it is not only an important disease-causing pathogen of fish and other cold blooded species, but also it is the etiological agent responsible for a variety of infectious complications in humans [2], [3]. As *Aeromonas* encompasses a diversity of strains or genotypes with varying virulence, it is often necessary to compare the degree of virulence of individual bacterial strains in order to assess the precise role and relative importance of various pathogenic mechanisms. To achieve this, a host must be chosen to evaluate and quantify the pathogenic potential of *Aeromonas*.

Intraperitoneal injection of mice with bacteria is useful in assessing the virulence of some bacteria, especially when combined with immune function modulation and evaluation of the dose that is lethal for 50% of the population (LD_50_) [4]. However, mice are not appropriate hosts for pathogens such as *Aeromonas* as these normally infect cold-blooded species living at low temperatures [5]. Recently, it has been reported that zebrafish is an excellent model for assessing the LD_50_ of *Aeromonas hydrophila* strains [6] and for examining host immune responses [7], but this model is not suitable for studying host-pathogen interactions at the molecular level [8]. In addition, using a vertebrate host to evaluate the virulence of bacteria is prohibitively time consuming, individually different, ethically problematic and expensive because large numbers of animals are required. In contrast, invertebrate model hosts represent a tempting alternative, as they not only address the defects mentioned above, but they are also associated with several important advantages, including simple immune systems, genetic tractability, and amenity to high-throughput experiments.

A number of different model systems, including amoeba [5], nematodes [9] and insects [10], have been introduced, and distinct model systems are more or less suitable for each particular pathogen. The *Dictyostelium* amoeba has been used as an alternative host to evaluate *Aeromonas* virulence [5]. However, Benghezal *et al*. [11] reported that *Tetrahymena* was more suitable than the amoeba model in a high-throughput screening study to identify inhibitors of *Klebsiella pneumoniae* virulence.

*Tetrahymena thermophila* is a free-living unicellular eukaryote that can be grown easily in cheap culture media over a wide range of temperature between 12°C to 41°C without the need for a CO_2_-enriched atmosphere. In addition, these cells are amenable to genetic analysis, most notably due to the small, fully sequenced genome, which allows for the elucidation of molecular interactions between host and pathogen [12], [13].

In the last decade, a lot of investigations have been performed on the interactions between *Tetrahymena* and certain bacteria, such as *Yersinia pestis* [14], [15], *K. pneumoniae* [11], *Escherichia coli* [16]–[18] and *Vibrio fischeri* [19]. A previous study in our laboratory described the interactions between *A. hydrophila* and *T. thermophila*, and indicated the potential of *Tetrahymena* as a permissive model to examine virulence of *A. hydrophila* [20].

The aim of this present study is to determine the correlation between the *Tetrahymena*–*Aeromonas* model system and the well-established animal model for assessing virulence of *Aeromonas* spp., in order to verify the reliability and accuracy of the former in evaluating bacterial virulence.

## Results

The virulence of 39 *Aeromonas* strains was assessed by determining LD_50_ values in zebrafish, and LD_50_s of all strains in zebrafish ranged from 1.3×10^2^ to 3.0×10^7^ (Table 1). Most dying fish showed clinical signs typical of hemorrhagic septicemia. Colonies of the 39 *Aeromonas* strains were recovered from infected fish, and no evident external injuries were observed in the surviving fish. According to the degree of virulence described by Pu *et al*. [21], 18 strains were included in the virulent category (LD_50_s of 10^2^−10^5^) while the remaining 21 strains were classified as avirulent (LD_50_s >10^6^).

**Table 1:** Virulence of 39 *Aeromonas* strains assessed in zebrafish and *T. thermophila* models.

Strain	*Aeromonas*species	Source	Sequenceof *gyr*B	LD_50_	Relative survivalOf *Aeromonas* (%)	Relative survival of*T. thermophila* (%)
NJ-35	*A. hydrophila*	Diseased Crucian carp	JX025789	1.3×10^2^	82.0±3.5	16.2±1.4
XY-16	*A. hydrophila*	Diseased Crucian carp	JX025797	5.8×10^2^	85.4±5.9	14.4±1.3
NJ-34	*A. hydrophila*	Diseased Crucian carp	JX025788	8.1×10^2^	78.3±3.9	19.1±0.8
XX-52	*A. hydrophila*	Diseased Crucian carp	JX025794	1.3×10^3^	75.6±1.3	17.1±1.4
BSK-10	*A. hydrophila*	Diseased Silver carp	JX413511	2.5×10^3^	73.0±3.8	27.3±3.8
CS-43	*A. hydrophila*	Diseased Silver carp	JX025780	2.6×10^3^	67.3±2.8	23.3±2.4
XX-49	*A. hydrophila*	Diseased Crucian carp	JX025793	6.3×10^3^	74.6±3.0	26.0±5.6
XX-58	*A. hydrophila*	Diseased Silver carp	JX025795	7.5×10^3^	61.1±6.6	23.3±3.0
XX-22	*A. hydrophila*	Diseased Common carp	JX025792	1.3×10^4^	65.5±2.7	33.4±4.9
NJ-1	*A. hydrophila*	Healthy Crucian carp	JX025785	1.7×10^4^	67.0±3.1	32.0±4.9
XX-62	*A. hydrophila*	Diseased Silver carp	JX025796	2.1×10^4^	54.0±2.8	28.0±5.8
NJ-24	*A. bestiarum*	Water	JQ815386	3.9×10^4^	62.2±5.3	31.7±3.3
XX-14	*A. hydrophila*	Diseased Silver carp	JX025791	4.9×10^4^	66.2±4.8	29.0±2.7
CS-2	*A. salmonicida*	Water	JX025811	1.5×10^5^	57.6±0.6	25.8±4.7
XH-16	*A. veronii*	Healthy Crucian carp	JX025900	3.4×10^5^	59.6±6.7	37.0±5.4
NJ-37	*A. hydrophila*	Diseased Eel	JQ815377	4.3×10^5^	63.1±5.1	42.0±4.9
GY-6	*A. veronii*	Healthy Common carp	JX025876	4.6×10^5^	56.0±1.4	39.2±1.2
J-1	*A. hydrophila*	Diseased Crucian carp	JX401926	8.5×10^5^	63.4±6.9	35.4±6.9
NJ-28	*A. hydrophila*	Diseased Crucian carp	JX025787	1.0×10^6^	48.2±4.6	38.5±6.2
NJ-16	*A. veronii*	Water	JX025903	1.2×10^6^	39.6±4.6	46.1±2.4
XH-17	*A. veronii*	Healthy Crucian carp	JX025899	1.2×10^6^	38.6±5.7	51.0±2.4
JH-19	*A. hydrophila*	Healthy Chinese bream	JX025784	1.3×10^6^	38.8±5.6	47.9±9.1
NJ-3	*A. hydrophila*	Water	JX025786	1.8×10^6^	52.0±2.6	49.0±3.9
XY-20	*A. veronii*	Healthy Crucian carp	JX025951	2.6×10^6^	27.1±4.7	58.6±7.4
XY-7	*A. veronii*	Healthy Grass carp	JX025956	2.9×10^6^	44.6±5.5	51.2±6.3
CS-51	*A. veronii*	Water	JX025863	3.1×10^6^	35.6±5.1	46.0±2.9
JH-2	*A. veronii*	Healthy Black carp	JX025890	4.8×10^6^	31.7±2.7	61.7±5.3
CS-16	*A. veronii*	Healthy Chinese bream	JX025854	4.8×10^6^	38.5±2.8	50.0±5.6
NJ-7	*A. media*	Water	JX025839	5.4×10^6^	23.5±5.4	71.0±2.7
XH-14	*A. veronii*	Healthy Crucian carp	JX025901	5.4×10^6^	14.2±2.2	68.3±4.2
GY-13	*A. veronii*	Healthy Common carp	JX025875	5.8×10^6^	22.8±5.0	59.9±4.8
CS-14	*A. veronii*	Diseased Chines bream	JX025843	6.2×10^6^	30.9±2.8	63.8±6.9
GY-23	*A. hydrophila*	Healthy Crucian carp	JX025782	6.7×10^6^	34.5±5.0	57.0±5.2
NJ-8	*A. media*	Water	JX025838	9.8×10^6^	10.8±1.8	81.2±5.5
NJ-21	*A. media*	Water	JX025837	1.1×10^7^	19.5±3.1	72.0±5.2
CS-34	*A. hydrophila*	Healthy Crucian carp	JX025779	1.3×10^7^	18.2±1.5	72.5±5.9
JH-17	*A. hydrophila*	Healthy Chinese bream	JX025783	2.0×10^7^	31.0±1.2	62.0±7.2
NJ-25	*A. media*	Water	JX025836	2.6×10^7^	22.2±0.8	76.7±5.1
CS-15	*A. veronii*	Diseased Chines bream	JX025842	3.0×10^7^	13.4±1.1	77.6±4.1

Relative survival of each bacterium at 12 h is expressed as the number of *Aeromonas* CFU grown in the presence of *T. thermophila* relative to the number of *Aeromonas* CFU cultivated alone. Relative survival of *T. thermophila* at 12 h is expressed as the number of *T. thermophila* cells co-cultured with *Aeromonas* spp. relative to the number of *T. thermophila* cells cultured alone.

**Figure 1:**
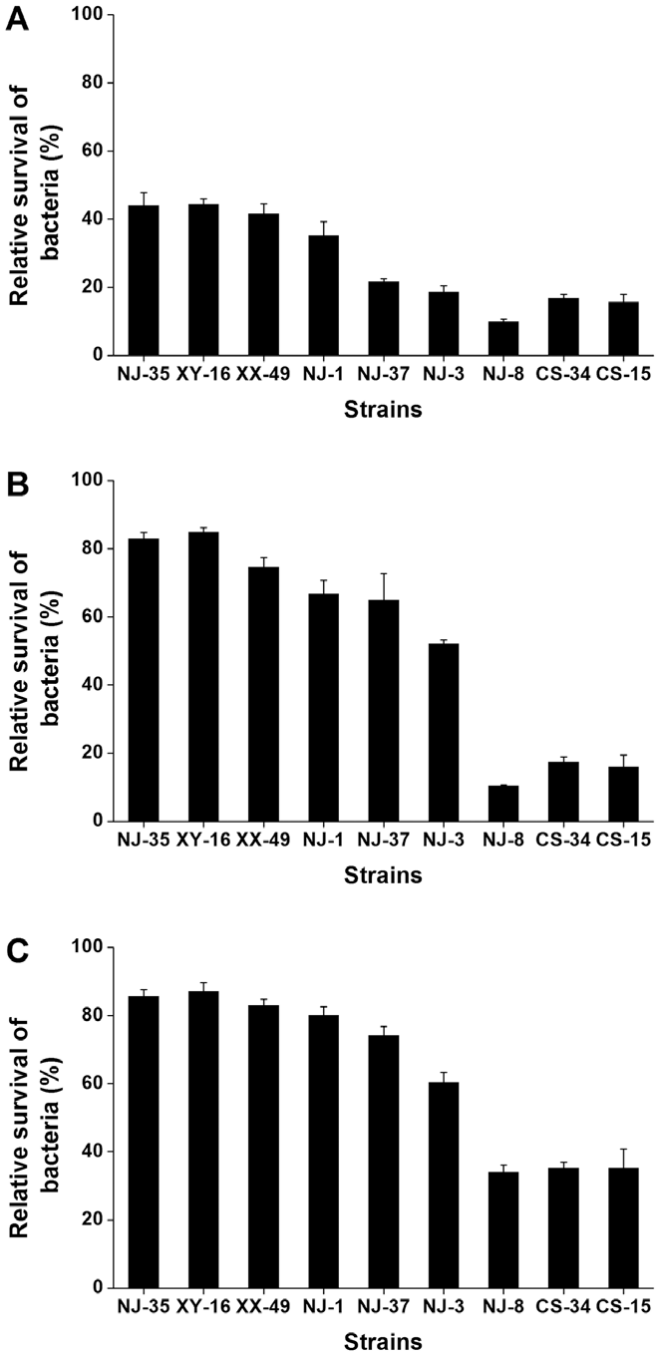
Relative survival of nine *Aeromonas* strains co-cultured with *T. thermophila* in three different proportions. Relative survival of each bacterium at 12 h is expressed as the number of *Aeromonas* CFU in the co-culture with *T. thermophila* relative to the number of *Aeromonas* CFU grown alone. At the start of the experiment, *T. thermophila* was at 1×10^5^ cells/ml while the *Aeromonas* spp. was at 1×10^8^ CFU/ml (A), 5×10^8^ CFU/ml (B) and 1×10^9^ CFU/ml (C), respectively. Error bars represent standard deviations from four independent experiments, with each experiment being comprised of four individual measurements.

To obtain the most appropriate relative population size of bacteria to protozoa, we determined the relative survival of nine *Aeromonas* strains with differential virulence after co-culture with *T. thermophila* for 12 h in three proportions. As shown in Fig. 1, *Aeromonas* strains that had greater virulence also had higher relative survival after co-culture studies, which suggest that it is possible to use relative survival to evaluate bacterial virulence. Although *T. thermophila* could inhibit the growth of the *Aeromonas* strains, the extent of the relative survival of *Aeromonas* was very different when they were co-cultured in different proportions with *T. thermophila*. The relative survival of *Aeromonas* strains inoculated at 1×10^9^ CFU/ml (Fig. 1C) was obviously greater than strains inoculated at the other two concentrations (Fig. 1A and Fig. 1B), and lowest relative survival was 34.0%. On the contrary, the *Aeromonas* strains inoculated at 1×10^8^ CFU/ml had lower relative survival, and greatest relative survival was 44.5% (Fig. 1A). This result shows that the initial *Aeromonas* concentration of 5×10^8^ CFU/ml gave the widest range of relative survival values (from 10.5% to 85.0%; Fig. 1B), and therefore was better for distinguishing *Aeromonas* strains of different virulence.

**Figure 2:**
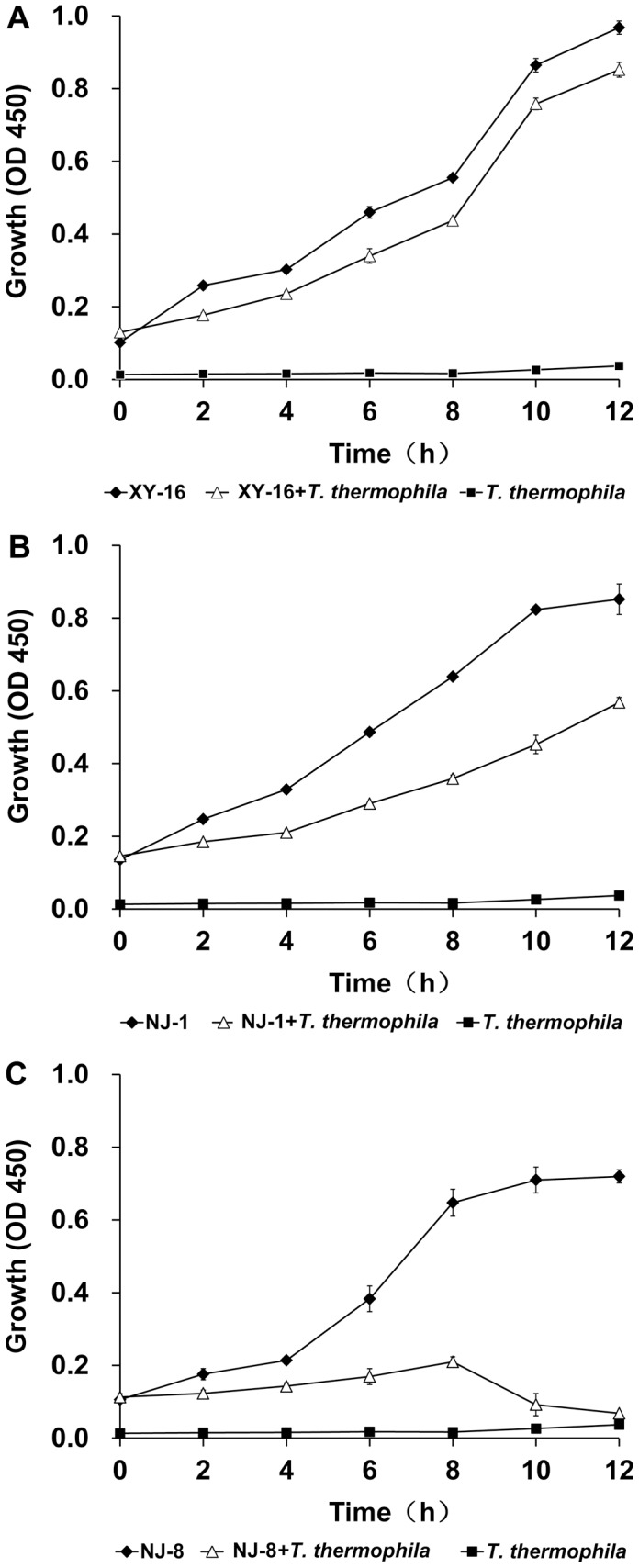
Growth of *A. hydrophila* XY-16 (A), *A. hydrophila* NJ-1 (B) and *A. media* NJ-8 (C) co-cultured in the presence or absence of *T. thermophila.* Bacterial growth was determined by measuring the absorbance at 450 nm (A_450_) during 12 h and (*T. thermophila* cells had only a negligible effect on A_450_). Data are expressed as the mean ± SD of four observations per time point.

To further investigate the effect of *T. thermophila* on the growth of *Aeromonas* strains of differential virulence, 5,000∶1 co-cultures of *Aeromonas* (5×10^8^ CFU/ml) and *T. thermophila* (1×10^5^ cells/ml) were used and growth dynamics of the *Aeromonas* strains were measured after co-culturing for 12 h. Bacterial growth was determined by measuring absorbance at 450 nm every 2 h. The growth dynamics of each strain incubated in the presence of *T. thermophila* could be used to divide the bacterial isolates into three categories (Fig. 2). *A. hydrophila* NJ-35, XY-16 and XX-49 formed the first category and *A. hydrophila* XY-16, which had an LD_50_ of 5.8×10^2^, was used as an example (Fig. 2A). In this category, co-culture with *T. thermophila* inhibited the growth of the *Aeromonas* strains but only very slightly, and these strains grew almost as well as control cultures grown in the absence of *T. thermophila* (Fig. 2A), indicating that to some extent they overcame predation by *T. thermophila*. The growth responses of strains in the second category can be illustrated using *A. hydrophila* NJ-1 (Fig. 1B), which had an LD_50_ of 1.7×10^4^. In this second category, the populations of the *Aeromonas* strains (*A. hydrophila* NJ-1, NJ-37 and NJ-3) always increased during co-culture, but the growth rate was significantly lower than observed in control incubations where these bacteria were cultured alone. In the third category is *A. hydrophila* CS-34, *A. veronii* CS-15, and *A. media* NJ-8, an avirulent strain with an LD_50_ of 9.8×10^6^ (Fig. 1C). The principal character of strains in this category is that the biomass of the *Aeromonas* cultured in the presence of *T. thermophila* either declined throughout the incubation period, or reached a peak before decreasing. These findings show that virulent *Aeromonas* strains were generally preyed upon less by *T. thermophila* compared to avirulent strains when cultured in the presence of *T. thermophila*. This indicates that the ability of an *Aeromonas* strain to resist *T. thermophila*-mediated phagocytosis is related to its virulence. This suggests that the virulence of *Aeromonas* strains can be assessed in the *T. thermophila* model by detecting relative survival of the bacteria when the co-culture is inoculated with population sizes of bacteria and protozoa at 5×10^8^ CFU/ml and 1×105 cells/ml, respectively.

**Figure 3:**
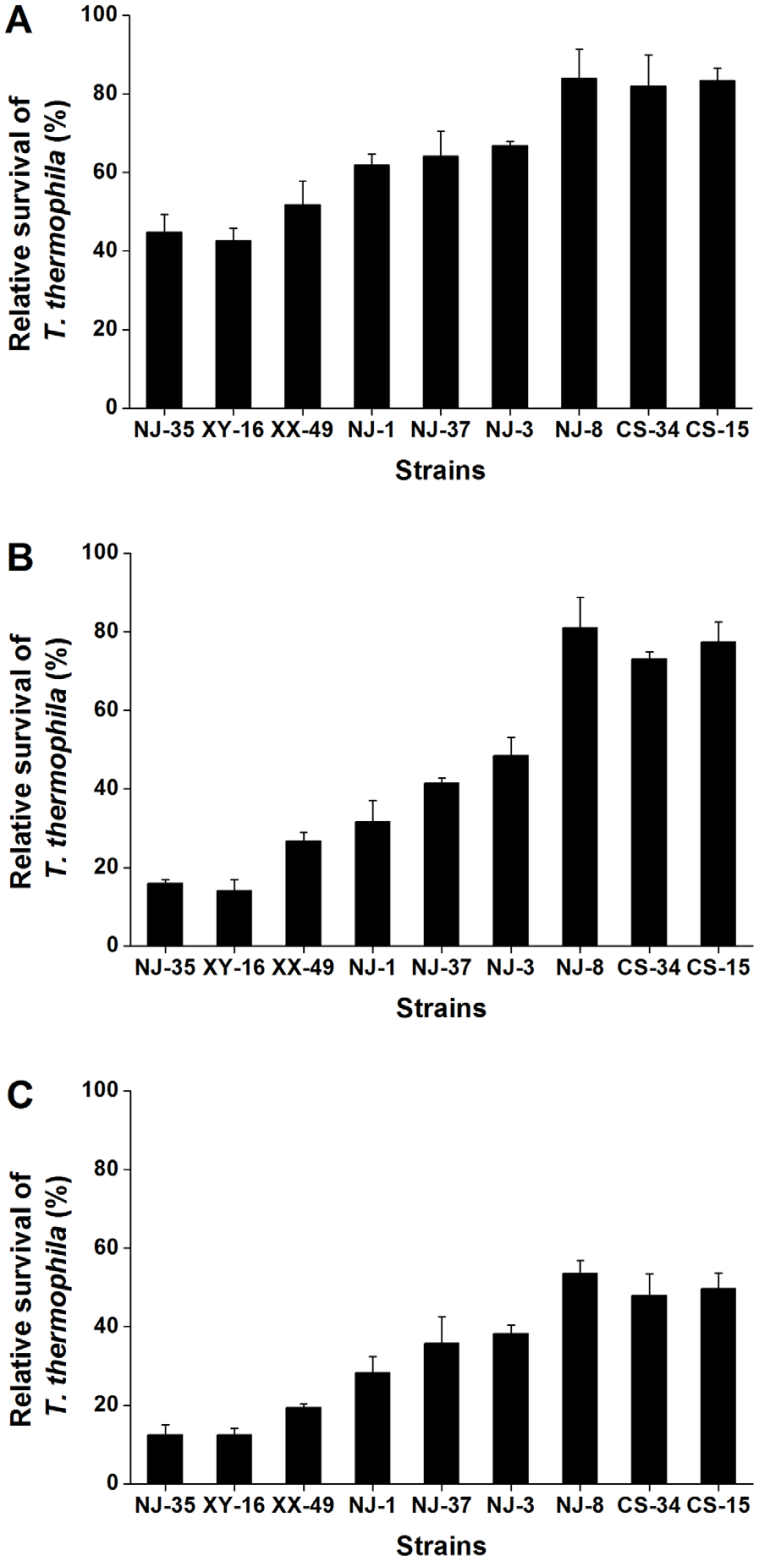
Relative survival of *T.thermophila* co-cultivated with *Aeromonas* strains in three different proportions. Relative survival of *T. thermophila* at 12 h is expressed as the number of *T. thermophila* cells in the co-culture with *Aeromonas* spp. relative to the number of *T. thermophila* cells cultured alone. At the beginning of the experiment, *T. thermophila* was at 2×10^5^ cells/ml while the *Aeromonas* spp. was at 2.0×10^8 ^CFU/ml (A), 1.0×10^9 ^CFU/ml (B) and 1.5×10^9 ^CFU/ml (C), respectively. Error bars represent SDs from a minimum of three independent experiments, with each experiment being comprised of six individual measurements.

From a protozoan perspective, relative survival of *T. thermophila* after co-culture with nine *Aeromonas* strains was determined. There was a tendency for increased relative survival of *T. thermophila* as *Aeromonas* virulence decreased (Fig. 3), regardless of the relative proportion of protozoan cells in the co-culture. It suggests that relative survival of *T. thermophila* after co-culture is related negatively to bacterial virulence. However, relative survival of *T. thermophila* was related to the initial concentrations of bacteria, such that when *T. thermophila* was mixed with *Aeromonas* strains at an initial density of 1.5×10^9^ CFU/ml, relative survival of *T. thermophila* was generally lower than 53.5% (Fig. 3C). In contrast, co-culture with *Aeromonas* strains at 2×10^8^ CFU/ml had a much smaller impact on *T. thermophila* viability, as relative survival of *T. thermophila* was greater than 42.6% in each case (Fig. 3A). Nevertheless, relative survival of *T. thermophila* after co-culture with *Aeromonas* at 1×10^9 ^CFU/ml ranged from 14.2% to 81.0% (Fig. 3B). Therefore, an initial minimal population of size of 1×10^9^ CFU/ml is able to provide better evidence at distinguishing *Aeromonas* strains of different virulence.

**Figure 4:**
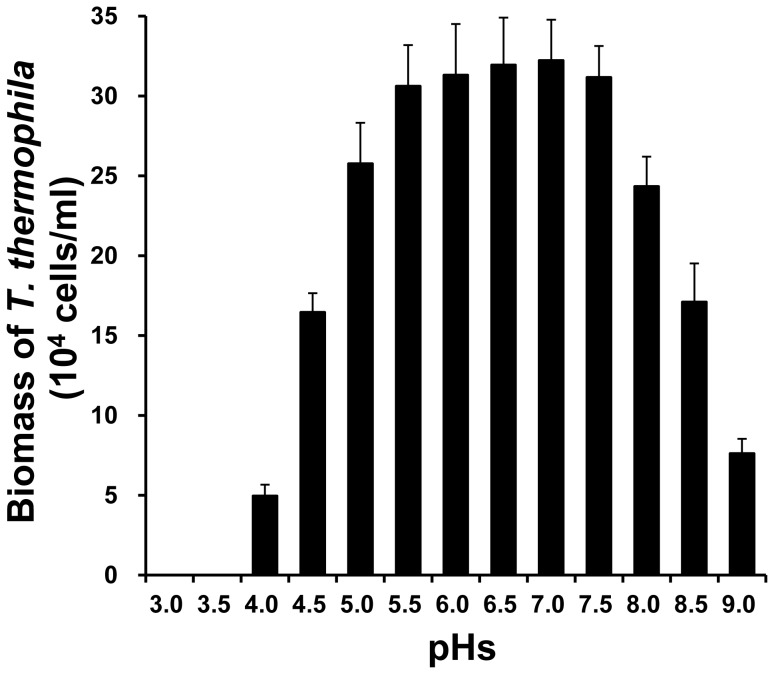
Effect of different pHs on the growth of *T. thermophila.* Biomass of *T. thermophila* was measured after cultured in different pH SPP medium for 12 h. Data are expressed as the mean ± SD of six measurements per time point.

Considering that pH changes by *Aeromonas* might affect the viability of *Tetrahymena*, we measured the pH values of medium in which *Aeromonas* were grown for 12 h and evaluated the influence of extracellular pH on *Tetrahymena* survival. The pH values of medium were maintained in the range 6.4 to 6.7 at 12 h after *Aeromonas* were cultured. The number of viable *Tetrahymena* was drastically decreased at very low (less than 4.5) or very high (greater than 8.5) pH values, and even decreased to zero at pH3.0 and pH3.5. But no significant changes were seen at the pH ranges between 5.5 and 7.5(Fig. 4).The result ruled out the possibility that pH changes over co-culture may kill *T. thermophila*, thus indicating the changes of relative survival of *T. thermophila* by pH conditions could be neglected.

**Figure 5:**
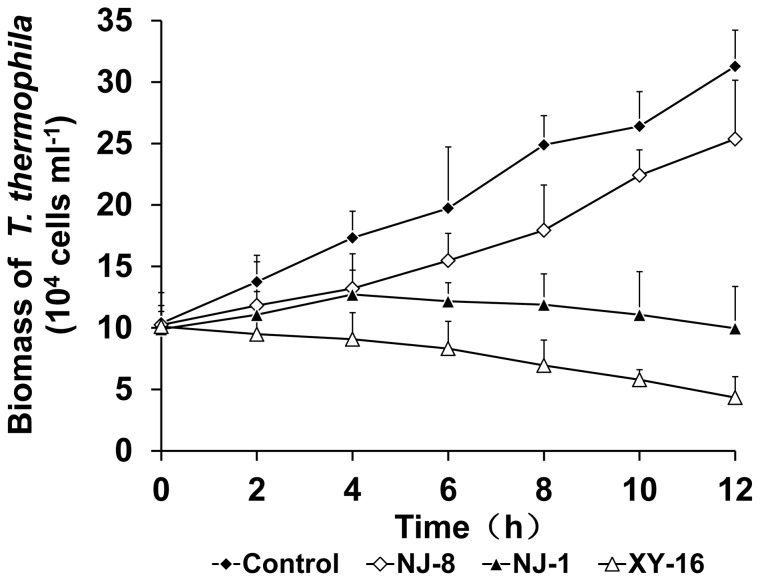
Growth of *T. thermophila* co-cultivated with *Aeromonas* isolates of different virulence. Growth of *T. thermophila* co-cultured with *A. hydrophila* XY-16, *A. hydrophila* NJ-1 and *A. media* NJ-8 was measured for 12 h after initiating the co-culture. The control group was *T. thermophila* grown alone in sterile SPP medium. Data are expressed as the mean +SD of six measurements per time point.

**Figure 6:**
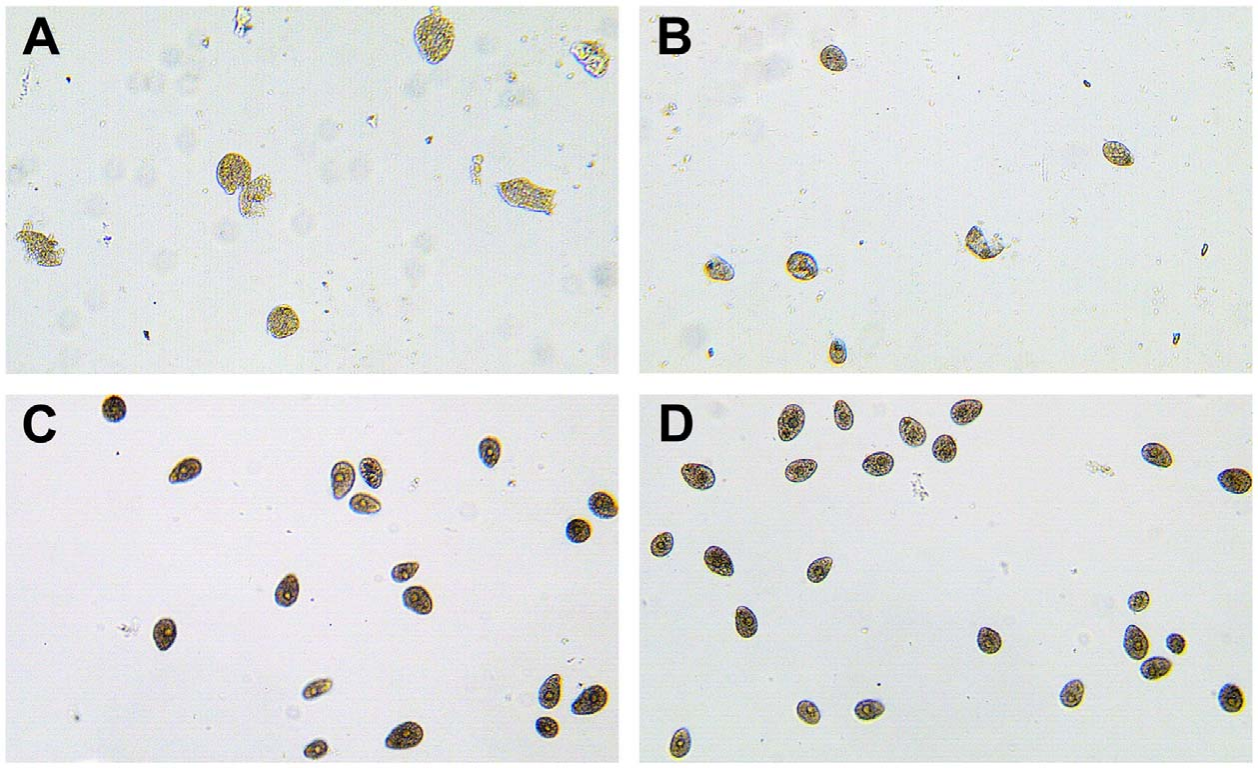
Morphological changes of *T. thermophila* cells after co-cultivation with different *Aeromonas* strains. *T. thermophila* was co-cultured with *A. hydrophila* XY-16 (A), *A. hydrophila* NJ-1 (B) and *A. media* NJ-8 (C) in SPP medium for 12 h before being observed under a light microscope. *T. thermophila* cultured in the absence of *A. hydrophila* (D) was used as a comparative control. Magnification × 100.

The effect of co-culture on growth of *T. thermophila* in a ratio of one cell to 5,000 *Aeromonas* cells was examined further. Biomass of *T. thermophila* cultured alone increased during the incubation and achieved a maximum concentration of 3.13×10^5^ cells/ml at 12 h. The growth of *T. thermophila* co-cultivated with three representative *Aeromonas* strains, *A. hydrophila* XY-16, *A. hydrophila* NJ-1 and *A. media* NJ-8, can be seen in Fig. 5. Co-culture with *A. hydrophila* XY-16 caused survival of *T. thermophila* to reduce persistently during 12 h. The number of *T. thermophila* cells grown in the presence of *A. hydrophila* NJ-1 increased slightly before 4 h but then decreased, while the number of *T. thermophila* cells co-cultured with *A. media* NJ-8 increased throughout the incubation period but at a rate lower than the controls. These data show that virulent *Aeromonas* strains exert negative effects on the growth of *T. thermophila* and even decrease the survival of *T. thermophila* remarkably, while avirulent *Aeromonas* isolates decrease growth rate but do not affect total *T. thermophila* biomass. The reduced growth of *T. thermophila* in the presence of virulent *Aeromonas* coincided with the death of *T. thermophila* cells after 12 h culture. From Fig. 6A and Fig. 6B it is observed that *T. thermophila* cells co-cultured with virulent *A. hydrophila* XY-16 and NJ-1 became deformed and even lysed leading to cell death. In contrast, *T. thermophila* co-cultured with avirulent *A. media* NJ-8 grew well, were in tact (Fig. 6C) and looked similar to cells in the control group (Fig. 6D). The growth of *T. thermophila* during co-culture showed that the virulence of *Aeromonas* strains can be assessed based on relative survival of *T. thermophila* when the relative population size was 1×10^9^ bacteria to 2×10^5^ protozoan cells.

On the basis of these co-culture conditions, the virulence of all 39 *Aeromonas* strains was evaluated further in the *T. thermophila* model. The relative survival of the *Aeromonas* strains at 12 h was calculated to identify any correlation with LD_50_s determined in the zebrafish. In general, relative survival of the strains decreased as the LD_50_s increased. The Pearson correlation coefficient between relative survival of *Aeromonas* and the LD_50_s of these strains in zebrafish was −0.683 (P<0.01); thus, the correlation is categorized as moderate according to Munro [22]. The resulting regression equation to calculate relative survival is as follows:

Y = 119.1 - 13.0 lgX (X is the LD_50_ of the *Aeromonas* isolate and Y is relative survival of the *Aeromonas* isolate).

**Figure 7:**
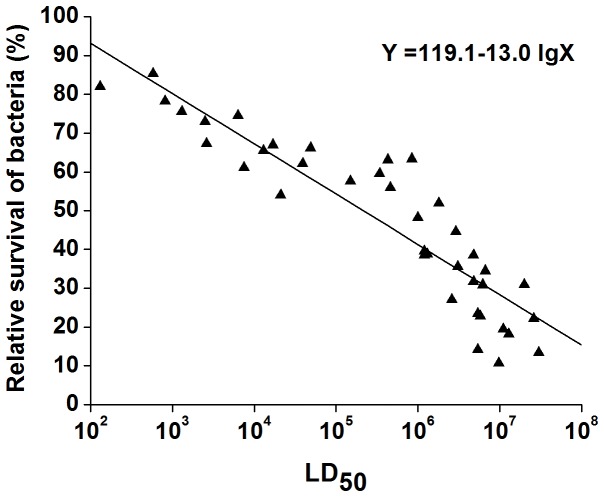
Linear regression model to predict relative survival of *Aeromonas* isolates from LD_50_ values. Concentrations of *Aeromonas* and *T. thermophila* cells at the start of the experiment were 5×10^8^ CFU/ml and 1×10^5^ cells/ml, respectively. LD_50_s of 39 *Aeromonas* isolates in zebrafish were used as the abscissa and a trend line was added. Each experiment was repeated a minimum of four times. Data presented represent the mean of a four replicates.

The standard deviation (SD) was 8.9, implying a prediction margin of ±SD. From the regression equation it is possible to predict that if the LD_50_ is above 1.0×10^6^, then relative survival of the bacterium would be below 41.1%±8.9%. Relative survival values of the *Aeromonas* strains were all greater than 40% when the LD_50_s were lower than 1.0×10^6^. Moreover, for most of the *Aeromonas* strains relative survival values were lower than 40% when the LD_50_s were above 1.0×10^6^ (Fig. 7), except for three strains (NJ-28, NJ-3, XY-7). For avirulent *Aeromonas* isolates there was no relative survival value greater than 60%, while there was no relative survival value of virulent *Aeromonas* isolates less than 40%. This suggests that an *Aeromonas* isolate could be determined as virulent if relative *Aeromonas* survival was above 60% or, on the contrary, avirulent if relative survival was below 40%.

**Figure 8:**
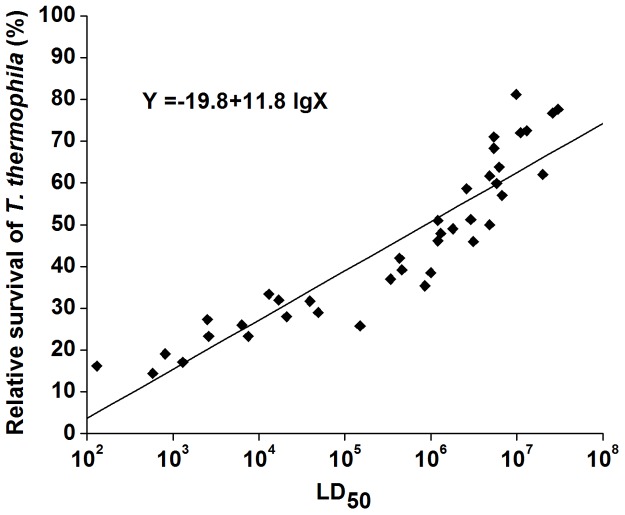
Linear regression model to predict relative survival of *T. thermophila* from LD_50_ values of the *Aeromonas* isolates. Concentrations of *Aeromonas* and *T. thermophila* cells at the start of the experiment were 1×10^9^ cells/ml and 2×10^5^ cells/ml, respectively. LD_50_s of 39 *Aeromonas* isolates in zebrafish were used as the abscissa and a trend line was added. Each experiment was repeated a minimum of six times per time point. Data presented represent the mean of a four replicates.

Relative survival of *T. thermophila* grown in the presence of the 39 *Aeromonas* strains increased as the LD_50_s of the *Aeromonas* strains increased (Fig. 8). This result indicates that there is a positive correlation between relative survival of *T. thermophila* and LD_50_ values in zebrafish since the Pearson correlation coefficient was 0.743 (*P*<0.01). The regression equation to calculate relative survival is as follows:

Y = -19.8+11.8 lgX (X is the LD_50_ of the *Aeromonas* isolate and Y is relative survival of *T. thermophila*).

The SD was 7.9, implying a prediction margin of ±SD. Thus, from the regression equation it is estimated that if the LD_50_ is above 1.0×10^6^, then relative survival of the bacteria would be greater than 51% ±7.9%.

The results show that almost all of the relative survival values of *T. thermophila* were lower than 40% when cultured in the presence of virulent *Aeromonas* isolates (LD_50_ values below 1.0×10^6^), except for *A. hydrophila* NJ-37. On the contrary, relative survival of *T. thermophila* was above 40% when co-cultured with avirulent *Aeromonas* isolates (LD_50_s of greater than 1.0×10^6^), except for *A. hydrophila* NJ-28. There was no relative survival value greater than 50% for *T. thermophila* co-cultured with a virulent *Aeromonas* isolate, while relative survival of *T. thermophila* was lower than 40% when co-cultured with avirulent isolates, except for *A. hydrophila* NJ28. Therefore, if relative survival of *T. thermophila* is greater than 50% after co-culture with an *Aeromonas* isolate, then this *Aeromonas* isolate is classified as avirulent. Conversely, the *Aeromonas* isolate is considered virulent if relative survival of *T. thermophila* in co-culture is less than 40%.

## Discussion

Members of the genus *Aeromonas* are distributed widely in aquatic environments [23], [24]. *Tetrahymena* is a fresh-water protozoan that is ecologically highly successful. Thus, it is likely that these two organisms confront each other in the natural environment. *Tetrahymena* can predate other microorganisms and it uses phagocytosis to ingest and degrade these cells [25]; however, it seems that certain bacterial virulence factors have evolved to defend against phagocytosis by *Tetrahymena* [18], [26]. Therefore, it is not surprising that many bacterial virulence mechanisms are active against *Tetrahymena* and, for example, Ly and Muller [27] showed that hemolytic *Listeria monocytogenes* induces lysis of *Tetrahymena pyriformis*, while only a few protozoa underwent lysis in the presence of nonhemolytic *Listeria innocua*. Previous work by this group with *A. hydrophila* J-1 and NJ-4 showed that *T. thermophila* could distinguish virulent and avirulent *A. hydrophila* strains [20]. In this present study, we attempted to correlate data from an animal infection model with a less complex and more ethically acceptable *Tetrahymena* infection model.

Considering animal models remain the gold standard for bacterial virulence testing [28], we first determined LD_50_s of 39 *Aeromonas* strains in zebrafish. The LD_50_s ranged from 1.3×10^2^ to 3.0×10^7^, implying that these strains were differentially virulent and could therefore be used to study the *Tetrahymena*-*Aeromonas* infection model. Afterwards, nine *Aeromonas* strains of differential virulence were selected to determine the most appropriate experimental conditions useful for evaluating *Aeromonas* virulence in the *Tetrahymena* model. This present study demonstrates that the relative population sizes of the bacterium/protozoan play key roles in virulence interactions. When relative population sizes of bacteria to protozoan were sufficiently high (5×10^8 ^CFU/ml bacteria to 1×10^5^ cell/ml protozoa or 1×10^9^ CFU/ml bacteria to 2×10^5^ cell/ml protozoa), it was more suited to distinguishing *Aeromonas* strains of different virulence.

High virulence *Aeromonas* strains could still grow in the presence of *T. thermophila*, while avirulent strains were largely phagocytozed. Moreover, *T. thermophila* grew well and cells were morphologically complete when co-cultivated with most avirulent *Aeromonas* strains; however, *T. thermophila* always became deformed and even lysed after co-culture with *Aeromonas* strains of high virulence. These results are in agreement with a previous report [20] and support the idea that resistance of bacteria to phagocytosis by *T. thermophila* correlates with virulence [11], [15]. Also, the virulence of an *Aeromonas* strain may be extrapolated from the ability of the protozoan to survive and grow in the presence of the bacterium.

To further support the idea that *T. thermophila* could be used reliably to evaluate *Aeromonas* virulence, the virulence of 39 *Aeromonas* strains was assessed in two ways using the *T. thermophila* model. From a bacterial perspective, an *Aeromonas* isolate could be determined as virulent when its relative survival in co-culture was above 60%, while the *Aeromonas* isolate was identified as avirulent when its relative survival was below 40%. This method is reliable for assessing *Aeromonas* virulence and discriminating virulent from avirulent strains. Unfortunately, we could not assess reliably the virulence of a few *Aeromonas* strains that had LD_50_ values between 1.0×10^5^ and 1.0×10^6^ because relative survival fluctuated from 60 to 40%. From a protozoan perspective, there were significant differences between the relative survival of *T. thermophila* co-cultured with virulent and avirulent *Aeromonas* strains. When relative survival of *T. thermophila* was lower than 40% in co-culture, the *Aeromonas* strain was regarded as virulent. In contrast, the strain was classified as avirulent if the relative survival of *T. thermophila* was greater than 50%. Hence, this implies that it is possible to evaluate the virulence of *Aeromonas* isolates by determining relative survival of co-cultured *T. thermophila*. Disappointingly, it was not possible to classify *Aeromonas* isolates as virulent or avirulent accurately when relative survival of *T. thermophila* was between 40 and 50%. This scenario involved *Aeromonas* strains with LD_50_s between 1.0×10^5^ and 1.0×10^6^, and is due mainly to the differences in virulence being only slight. Nevertheless, virulence assessments in the *Tetrahymena*-*Aeromonas* co-culture model using these two approaches generally complemented each other, and there existed good correlation between results obtained in the *Tetrahymena* model and in zebrafish, demonstrating the usefulness of this model system as a novel tool for investigating virulence determinants in *Aeromonas* spp.

The mechanisms that determine bacterial resistance to grazing protozoan are not known. Our previous study [20] showed that the extracellular products by *Aeromonas* might contribute to the death of *T. thermophila*. But the correlation of resistance with known virulence genes was not fully understood. Further work is required to enable understanding of interactions between the bacterial extracellular factors and host at the cellular and molecular level.

In conclusion, this is the first study to demonstrate that *Tetrahymena* can be used as an alternative host model for specifically determining virulence of *Aeromonas* strains. This protozoan model is an inexpensive and sensitive for such investigations, and can facilitate the systematic evaluation of virulence in hundreds or thousands of bacterial strains, and so is helpful for high-throughput screening of bacterial virulence factors. Moreover, as *T. thermophila* is amenable to genetic analysis and the entire genome has been published [29], [30], this system might also allow analysis of host resistance mechanisms.

## Materials and Methods

### Ethics Statement

Animal experiments were carried out according to animal welfare standards and approved by the Ethical Committee for Animal Experiments of Nanjing Agricultural University, China.

### Strains, Media and Culture Conditions

A total of 39 *Aeromonas* strains were used in this study, including 20 *A. hydrophila*, 13 *Aeromonas veronii*, four *Aeromonas media*, one *Aeromonas bestiarum,* and one *Aeromonas salmonicida* (Table 1). All strains were identified by biochemical tests, polymerase chain reaction (PCR) amplification and sequencing of 16S rRNA and DNA gyrase subunit B (*gyrB*) genes. *T. thermophila* SB210 was obtained from Dr Miao Wei, Institute of Hydrobiology, Chinese Academy of Sciences.

*Aeromonas* strains were incubated in liquid Luria Bertani broth (LB) at 28°C. *T. thermophila* SB210 was grown axenically in SPP medium (2% proteose peptone, 0.1% yeast extract, 0.2% glucose, 0.003% sequestrene) at 30°C.

### LD_50_ Determinations

LD_50_ values of the 39 *Aeromonas* strains were determined in a zebrafish model. Eighty-day-old AB line inbred zebrafish weighing about 3g were bought from the Pearl River Fishery Research Institute, Chinese Academic of Fishery Science, and allowed to acclimate for at least 3 days before use. Single colonies of each bacterial strain were inoculated into LB and then overnight cultures were diluted 1∶100 in fresh LB. The inoculated cultures were incubated at 28°C and harvested during logarithmic phase of growth when the optical density at 600 nm reached 0.6. Harvested *Aeromonas* cells were washed twice in sterile phosphate-buffered saline (PBS), and then resuspended in PBS to appropriate concentrations. For each *Aeromonas* isolate, eight groups of ten fish were intraperitoneally injected with 0.02 ml of serially tenfold diluted bacterial suspensions containing 10^1^–10^8^ CFU. Another ten zebrafish (the control group) were injected intraperitoneally with 0.02 ml sterile PBS only. Zebrafish were placed in tanks, one for each bacterial infection group. Tanks were aerated and non-circulating, and were maintained at 28°C. The zebrafish were observed until one week post infection. Water was occasionally added to account for evaporation and any dead fish were removed from the tanks during the course of the experiments. For each determination, three separate experiments were performed in which ten fish were infected with each concentration of *Aeromonas*. Three replicate tanks of fish were used in the challenge tests. Survival data were analyzed by the method of Reed and Muench [31] for the calculation of LD_50_ values.

### Effect of Co-culture on *Aeromonas* Growth

*T. thermophila* SB210 was cultured in sterile SPP medium at 30°C [18], [20] for 36 h until stationary phase of growth using an initial inoculum of 10^3^ cells/ml. Cells in this culture were harvested by centrifugation at 2,000 g for 10 min at 15°C [20] with the consideration of keeping the normal morphology and athletic ability, washed twice with sterile SPP medium and adjusted to 1×10^5^ cells/ml. This density of *T. thermophila* was selected on the basis of a preliminary sighting study, in which the starting density was screened from the fixed levels of 1×10^5^ [11], 2×10^5^ and 3×10^5^ cells/ml as a density expected to show varying degrees of bacterial growth after co-cultured with *Aeromonas* strains of different virulence. Nine *Aeromonas* strains that had LD_50_s ranging between 1.3×10^2^ and 3.0×10^7^ in zebrafish were chosen by a stratified random sampling method [32] to be representative of differently virulent strains. Overnight cultures of these *Aeromonas* strains were diluted 100-fold in fresh LB and harvested by centrifugation at 4,000 g for 5 min after incubating at 28°C for 12 h. Harvested cultures were washed twice in sterile SPP medium and resuspended to 1×10^8^, 5×10^8^ and 1×10^9^ CFU/ml, respectively. Five-hundred microliters of suspensions from these nine *Aeromonas* strains were mixed with equal volumes of *T. thermophila* SB210 cells. Then, 100 µl of these mixed cell suspensions were transferred into wells of 96-well plates; controls contained the bacterial strains at their respective concentrations mixed with an equal volume of SPP medium only and, similarly, *T. thermophila* SB210 cell suspensions supplemented with an equal volume of SPP medium. Sterile SPP medium was used as the blank well. Plates were incubated for 12 h at 28°C, and bacterial growth was determined every 2 h by measuring changes in absorbance of the suspensions at 450 nm. *T. thermophila* cells only accounted for negligible absorbance [11]. Evaporation during the incubation period was avoided by using a constant temperature-humidity incubator. Relative survival of bacteria (%) was expressed as the number of cells remaining in culture at 12 h relative to the number of cells present at the start of the co-culture. Each measurement was repeated in quadruplicate.

### Effect of Co-culture on *T. thermophila* Viability

*T. thermophila* cells were harvested after being cultured at 30°C for 36 h, washed twice with sterile SPP medium and resuspended to 2×10^5^ cells/ml. Nine *Aeromonas* strains, representative of differently virulent strains, were inoculated into LB medium and harvested after 12 h culture. The concentrations of these strains were adjusted to 2×10^8^, 1×10^9^ and 1.5×10^9 ^CFU/ml with sterile SPP medium. Five hundred microliters of each of the *Aeromonas* strains at the different concentrations were added to a same volume of *T. thermophila* cells in suspension in 2 ml Eppendorf tubes, and incubated at 28°C without shaking. To control for the effect of adding bacteria to *T. thermophila* cell suspensions, *T. thermophila* cells were separately incubated with an equal volume of sterile SPP medium. Every 2 h, 100 µl of each mixture was removed from the Eppendorf tubes, fixed with iodine solution and used to count the number of *T. thermophila* cells using a hemacytometer. The cellular morphology of *T. thermophila* cells incubated for 12 h was examined under a light microscope (Olympus cx21) after they had been fixed with iodine solution. Relative survival of *T. thermophila* (%) was expressed as the number of cells remaining in culture at 12 h relative to the number of cells present at the start of the co-culture. Each measurement was performed six times per time point and then mean values were calculated. Each experiment was repeated at least four times.

To evaluate the influence of pH changes by *Aeromonas* on *T. thermophila* viability, one milliliter of each of the nine *Aeromonas* strains with above different concentrations were cultured in the SPP medium with an initial pH value of 7.0 at 28°C for 12 h without shaking, and then pH values of SPP medium were measured by a pH meter (inoLab pH 7300, Germany). In addition, one milliliter of *T. thermophila* at an initial density of 2×10^5^ cells/ml were cultured alone in the SPP medium with different pH values in the range 3.0 to 9.0 at 28°C for 12 h, and then *T. thermophila* were counted.

### Assessment of *Aeromonas* Virulence using the *T. thermophila* Model

The virulence of the 39 *Aeromonas* strains for which LD_50_s had been determined in zebrafish was assessed using the *Aeromonas-Tetrahymena* infection model in two ways. First, cultures of the 39 *Aeromonas* strains were harvested, washed twice in SPP medium and adjusted to 5×10^8^ CFU/ml, while *T. thermophila* cells were resuspended at 1×10^5^ cells/ml. Second, the *Aeromonas* cultures were resuspended at 1×10^9^ CFU/ml, while *T. thermophila* suspensions were adjusted to 2×10^5^ cells/ml. Then the relative survival of *T. thermophila* was determined after co-culturing. The Pearson correlation coefficient (*r*) was used to quantify the relationship between the relative survival values of *Aeromonas* or *T. thermophila* and the LD_50_s of *Aeromonas* isolates in zebrafish. Additionally, linear regression models were used to predict the relative survival values from the LD_50_s of *Aeromonas* isolates in zebrafish. Each experiment was repeated at least four times.

### Statistical Analysis

Pearson correlation coefficients were calculated using the SPSS v20.0 statistics software. The linear regression model was fitted using the Origin 7.5 software. Error bars presented in the figures represent standard deviations of the means of multiple (>3) replicate experiments. A *P*-value <0.05 was considered to indicate a significant difference.
